# Circadian rhythm response and its effect on photosynthetic characteristics of the *Lhcb* family genes in tea plant

**DOI:** 10.1186/s12870-024-04958-0

**Published:** 2024-04-25

**Authors:** Zhi-Hang Hu, Nan Zhang, Zhi-Yuan Qin, Jing-Wen Li, Jian-Ping Tao, Ni Yang, Yi Chen, Jie-Yu Kong, Wei Luo, Xuan Chen, Xing-Hui Li, Ai-Sheng Xiong, Jing Zhuang

**Affiliations:** 1 Tea Science Research Institute, College of Horticulture, Nanjing Agricultural University, Nanjing, 210095 China; 2 State Key Laboratory of Crop Genetics & Germplasm Enhancement and Utilization, College of Horticulture, Nanjing Agricultural University, 1 Weigang, Nanjing, 210095 China

**Keywords:** *Camellia sinensis*, *CsLhcb*, Circadian clock, Photosynthetic parameters, Gene expression

## Abstract

**Background:**

The circadian clock, also known as the circadian rhythm, is responsible for predicting daily and seasonal changes in the environment, and adjusting various physiological and developmental processes to the appropriate times during plant growth and development. The circadian clock controls the expression of the *Lhcb* gene, which encodes the chlorophyll a/b binding protein. However, the roles of the *Lhcb* gene in tea plant remain unclear.

**Results:**

In this study, a total of 16 *CsLhcb* genes were identified based on the tea plant genome, which were distributed on 8 chromosomes of the tea plant. The promoter regions of *CsLhcb* genes have a variety of *cis*-acting elements including hormonal, abiotic stress responses and light response elements. The *CsLhcb* family genes are involved in the light response process in tea plant. The photosynthetic parameter of tea leaves showed rhythmic changes during the two photoperiod periods (48 h). Stomata are basically open during the day and closed at night. Real-time quantitative PCR results showed that most of the *CsLhcb* family genes were highly expressed during the day, but were less expressed at night.

**Conclusions:**

Results indicated that *CsLhcb* genes were involved in the circadian clock process of tea plant, it also provided potential references for further understanding of the function of *CsLhcb* gene family in tea plant.

**Supplementary Information:**

The online version contains supplementary material available at 10.1186/s12870-024-04958-0.

## Background

The circadian rhythm, also known as the circadian clock, is an endogenous timekeeping mechanism with a cycle of about 24 h (the relative length of day and night) [1]. In most plants, it’s able to predict daily and seasonal changes in the surrounding environment and adjust various physiological and developmental processes to the optimal time period [2]. The phenomenon of circadian clock has been observed in many organisms, such as plants [3–6]; cyanobacteria [7]; animals [8], etc. Diurnal regulation of photoreceptors and gene expression have been extensively studied in flowering plant systems, particularly in *Arabidopsis thaliana*. At present, studies on tea plant mainly focus on secondary metabolism, such as theanine and lignin synthesis etc [9–11], and relatively few researches about circadian rhythm in tea plant.

The tea plant [*Camellia sinensis* (L.) O. Kuntze] is an evergreen woody plant in the genus *Camellia* L. in the Theaceae family that is widely grown worldwide, especially in China. According to statistics of tea association, tea planting area in China has reached 3.1 million hm^2^. As one of the most important factors for plant growth and development, light can affect plant photosynthesis, morphological construction and accumulation of secondary metabolite in the plant [12]. The utilization efficiency of photosynthesis is an important factor affecting the growth and development of tea plant. During photosynthesis, photosynthetic parameters and stomatal opening of leaves will reflect the photosynthetic capacity of tea plant. Du et al. analyzed the transcriptome of 2 poplar progeny (ZT4, ZT16, ZT22) with different photosynthetic rates at 3 time points, and found that plant hormone signal transduction and transcription factors-related genes increased leaf size and stomatal movement, indicating that many potential key regulatory factors were closely related to chlorophyll content and photosynthesis [13].

Circadian clocks are regulated by several genes. Key genes controlling circadian clocks include *Circadian clocks-associated 1* (*CCA1*), *Late elongated hypocotyl* (*LHY*), and *Pseudo response regulators* (*PRRs*) etc [14]. Initially, studies confirmed that CCA1 is a transcription factor binding to the promoter region of *Lhcb1.3* gene of *A. thaliana*. CCA1 protein is a key element in the function of the photo pigmentation signal transduction pathway, leading to increased transcription of this *Lhcb* gene in *A. thaliana* [15]. Photosynthesis is also regulated by circadian rhythms, and proper circadian rhythm is beneficial to plant growth and development [16]. The expression of the genes involved in the light-harvesting chlorophyll-protein complex is also regulated by the circadian clock [17, 18]. In *A. thaliana*, phytochrome is the main light signal receptor, which is an important component of light signal input to the circadian clock. Under different light conditions, *Lhcb* and *LHY* showed obvious circadian rhythm expression pattern. LHY is considered to be a transcription factor regulating the *Lhcb* gene, acting in or near the vibrator center of *A. thaliana* [19].

The light-harvesting chlorophyll a/b-binding proteins of photosystem II (LHCIIb, formerly CAB2) were regulated by photoreceptors at the transcriptional level. The *Lhcb* gene was one of the fastest light-responsive genes in the model system. In terms of rapid response time and sensitivity to light, both provide the most reliable indicators of the transcriptional capacity of plant systems [20]. Kellmann et al. used potatoes as test materials and found that the expression of *Lhcb* gene increased significantly at around noon, but decreased to an almost undetectable level at midnight [21]. In *A. thaliana* plants growing under various photoperiodic conditions, *Lhcb* gene expression levels declined from peak levels before dusk and increased from trough levels before dawn [22]. Circadian rhythms provided a higher competitive advantage for both plant growth and photosynthesis [23]. Stomatal opening and closing, which were mainly affected by light, was an important gateway for plants to exchange gas with the outside world. Stomatal opening was also another kind of circadian rhythm regulation to observe cellular level processes. Somers et al. found that the stomatal conductance of *A. thaliana* was higher during the day than at night [24]. Li et al. discovered and revealed the evolution and phylogeny of the *Lhcb* gene in Rosaceae and highlighted the key role of *Lhcb* in the pear cryogenic response [25]. At present, the molecular mechanism and action mechanism of *Lhcb* gene family in tea plant are still unclear.

Here, the members of *CsLhcb* gene family were identified based on genomic data of tea plant, and then, chromosome localization, evolutionary tree and conserved domain were analyzed. The transcriptome data of tea plants were used to analyze the gene expression profiles in different tissues and abiotic stress treatments. In order to study the change of circadian rhythm of ‘Baiyeyihao’ at intervals of 4 h within two photoperiod (48 h), we started lighting at 9:00 (the initial time was recorded as 0 h), took the first sample, sustained light for 12 h, and then darkness for 12 h, where time is the variable. Fluorescence quantitative PCR was used to detect the circadian rhythms response of *CsLhcb* gene family in ‘Baiyeyihao’ during the two photoperiod periods (48 h). At the same time, the photosynthetic parameters and stomatal opening of tea leaves at different time points were also detected and analyzed. This study provided reference for further research on the circadian rhythm mechanism and function of *CsLhcb* gene family in tea plant.

## Results

### Systematic evolution and gene structure analysis of *CsLhcb* gene family in tea plant

**Fig. 1:**
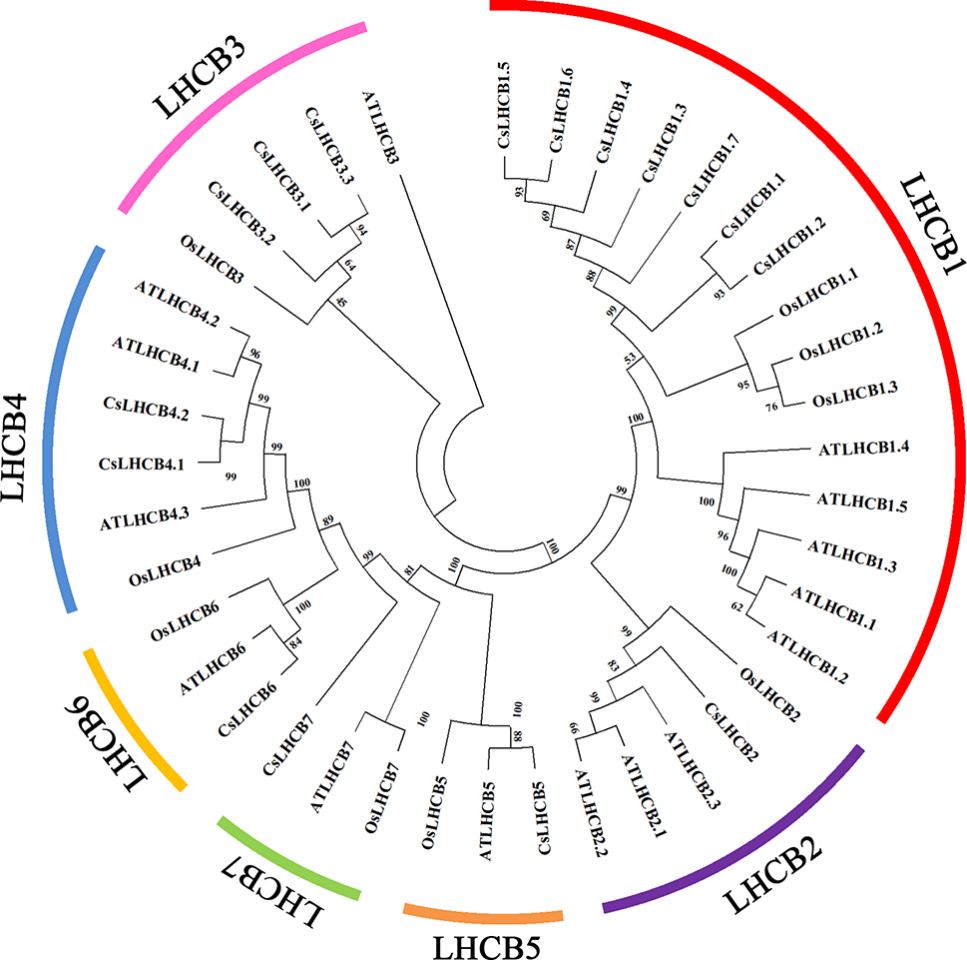
Phylogenetic analysis of CsLHCBs in *C. sinensis*

According to the reported nucleotide sequences of *Lhcb* genes in *A. thaliana* and rice, a total of 16 *CsLhcb* family genes were identified and obtained. The phylogenetic tree analysis of LHCB protein sequences of tea plant, *A. thaliana* and rice showed that CsLHCB proteins could be divided into 7 subgroups with homologous genes of *A. thaliana* and rice. They were CsLHCB1, CsLHCB2, CsLHCB3, CsLHCB4, CsLHCB5, CsLHCB6 and CsLHCB7, respectively. Each subpopulation contained 1 ∼ 7 CsLHCBs. LHCB1, LHCB2 and LHCB3 were the main body LHCII (or LHCIIb), accounting for 11/16 (Fig. 1).

### Analysis of gene structure and protein conserved motifs of tea plant

**Fig. 2:**
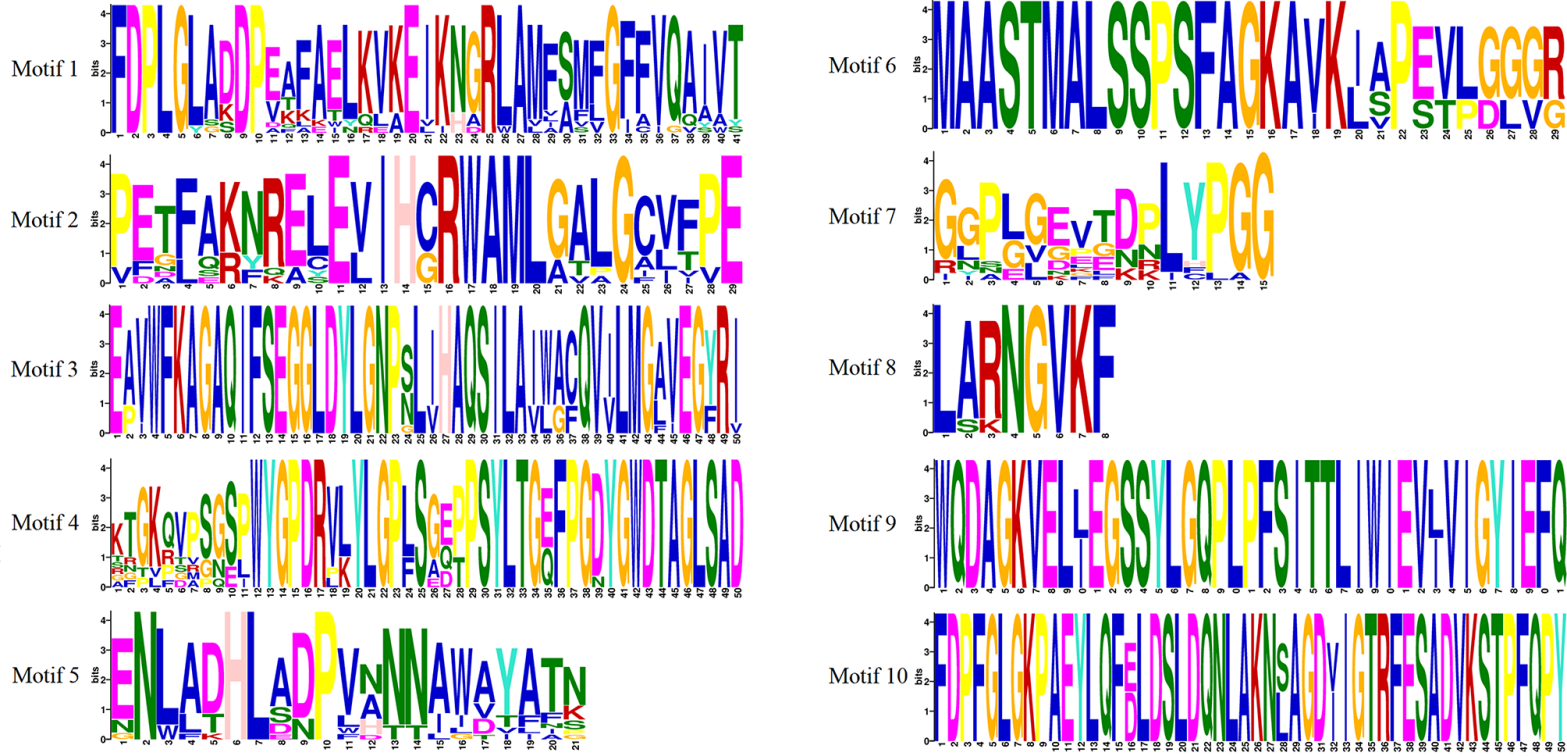
Conserved motif analysis of CsLHCBs in *C. sinensis* heights of residues within a stack indicate the relative frequency of each residue at that position

**Fig. 3:**
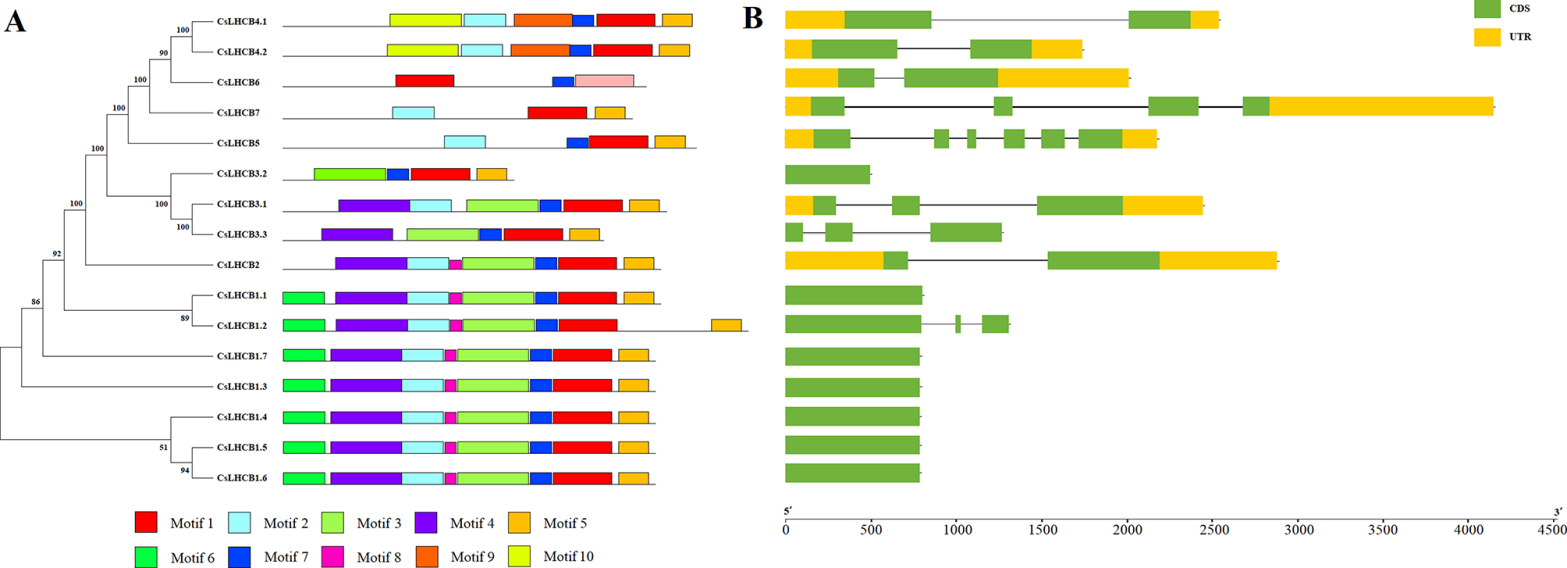
Structure and Conservative motifs of *CsLhcb* family genes of *C. sinensis ***A**: Conserved motifs of CsLHCB protein, Motif 1 to 10 represented different conserved motifs, represented by different colors **B**: Intron and exon structure diagram of *CsLhcb* genes, UTR represents non-coding region, CDS represents exon

MEME was used to predict and analyze the conserved motifs of CsLHCB protein in tea plant. A total of 10 conserved motifs were identified (Fig. 2). Motif 1, Motif 5 and Motif 7 appeared in CsLHCB family members in tea plant, indicating that these motifs were relatively conserved in the evolutionary process. CsLHCB1.1 ∼ CsLHCB1.7 had the same conservative motif, and CsLHCB4.1 and CsLHCB4.2 also had the same conservative motif. Motif 3 and Motif 6 were only found in three family members of LHCII b, suggesting that LHCII b was composed of similar conserved domains and might play a key role in evolution. In order to further understand the function of *CsLhcb* family genes of tea plant, TBtools was used to draw the gene structure map of *CsLhcb* for analysis (Fig. 3). The results showed that some regions of *CsLhcb2*, *CsLhcb3.1*, *CsLhcb4.1*, *CsLhcb4.2*, *CsLhcb5*, *CsLhcb6* and *CsLhcb7* could not translate proteins. The *CsLhcb* gene in the same subfamily was composed of similar gene structure and might be derived from the same gene.

### Chromosome mapping of *CsLhcb* family genes in tea plant

**Fig. 4:**
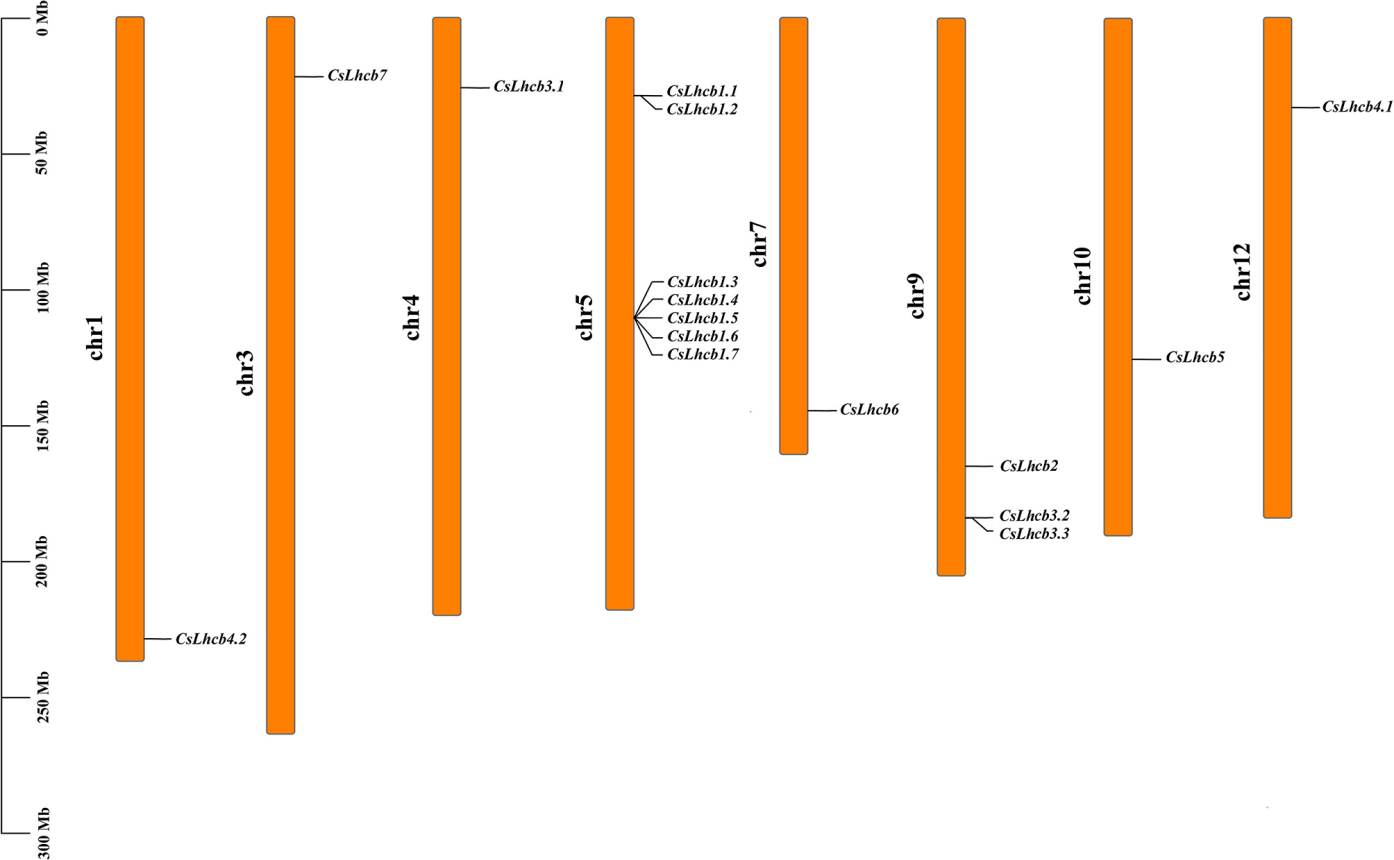
Chromosomal locations of *CsLhcb* family genes in *C. sinensis*

According to the chromosomal localization analysis of Shuchazao database [26], the results showed that 16 *CsLhcb* family genes were distributed on 8 chromosomes of tea plant, which were located on chromosomes 1, 3, 4, 5, 7, 9, 10 and 12, respectively. Three homologous gene clusters of *CsLhcb* gene were analyzed, namely *CsLhcb1.1* and *CsLhcb1.2*, which were located on chromosome 5; *CsLhcb1.3*, *CsLhcb1.4*, *CsLhcb1.5*, *CsLhcb1.6* and *CsLhcb1.7*; *CsLhcb3.2* and *CsLhcb3.3* were located on chromosome 9 (Fig. 4).

### Analysis of *cis*-acting elements of *CsLhcb* family genes in tea plant

The prediction analysis of *cis*-acting elements in the promoter region of *CsLhcb* genes in tea plant showed that Light Response Elements were all present in *CsLhcb* genes (Table 1). The results indicated that all members of *CsLhcb* family genes may be involved in the light reaction process. The circadian clock response elements were distributed in *CsLhcb1.1*, *CsLhcb1.2*, *CsLhcb3.2*, *CsLhcb3.3*, *CsLhcb6* and *CsLhcb7*, indicating that these 6 genes might be involved in the photoperiod of tea plant. At the same time, *CsLhcb* family genes was also may be involved in abiotic stress response and hormone response related *cis*-acting elements.

**Table 1:** Primers for RT-qPCR of *CsLhcbs* genes

Gene	Gene ID	Forward primer sequence (5’-3’)	Reverse primer sequence (5’→3’)
*CsLhcb1.1*	TEA001868.1	GGCAGAGGAAGGATCAGCAT	CACGGTTCTTGGCGAATGTT
*CsLhcb1.2*	TEA001864.1	CGTTGGCAGAGGAAGGATCA	CGAACTTGACACCATTGCGG
*CsLhcb1.3*	TEA019232.1	ATTGTCCCGGAGGTTCTTGG	GGGCTCACCGGATAATGGAC
*CsLhcb1.4*	TEA030366.1	GGTTCTTGGTGGTGGAAGGA	ATTGCCCAGTGAGGTAGGATG
*CsLhcb1.5*	TEA009770.1	GGTTCTTGGTGGTGGAAGGA	ATTGCCCAGTGAGGTAGGATG
*CsLhcb1.6*	TEA030368.1	GCCTAGCCGATGATCCAGAG	GGTCAGCCAGGTTCTCCAAT
*CsLhcb1.7*	TEA009793.1	GGACGGATCAGTATGAGGAAGA	GGAAGACGCAACCCAAAGC
*CsLhcb2*	TEA028092.1	GGCAGTTTGGTTCAAGGCTG	CCCATGAGCACTACTTGGCA
*CsLhcb3.1*	TEA017256.1	CAGACTCCGTCGTACCTCAC	GCCTCAGGATCAGCAGACAA
*CsLhcb3.2*	TEA001060.1	TGAGGGCTTTCGCATTAACG	TCAAGGTGGTCCAACAGGTT
*CsLhcb3.3*	TEA021966.1	GAGGGCTTCCGCATTAACG	GGTCCAACAGGTTCTCAAGTG
*CsLhcb4.1*	TEA026680.1	CTGCCGCCACATCATCATT	CGCCGAGTTCTTAGCCAAG
*CsLhcb4.2*	TEA032678.1	GCCGCCACTTCATCCTTCA	CAATGTTCCGTCCAACCACTC
*CsLhcb5*	TEA023017.1	ATCGCTGAAGTTGTTCTTGTTG	AGTCTACCGTTCTTGATCTCCT
*CsLhcb6*	TEA030284.1	TGCTGGCTGCTGCTCTTAA	CTCTGTACCACTTGAGGAATGC
*CsLhcb7*	TEA008963.1	CGTCAGTCCTTTCCTCTTCCA	GCATCCAGTCAGCCGTCAT
*GAPDH*		TTGGCATCGTTGAGGGTCT	CAGTGGGAACACGGAAAGC

### Analysis of *CsLhcb* family genes expression pattern in tea plant

**Fig. 5:**
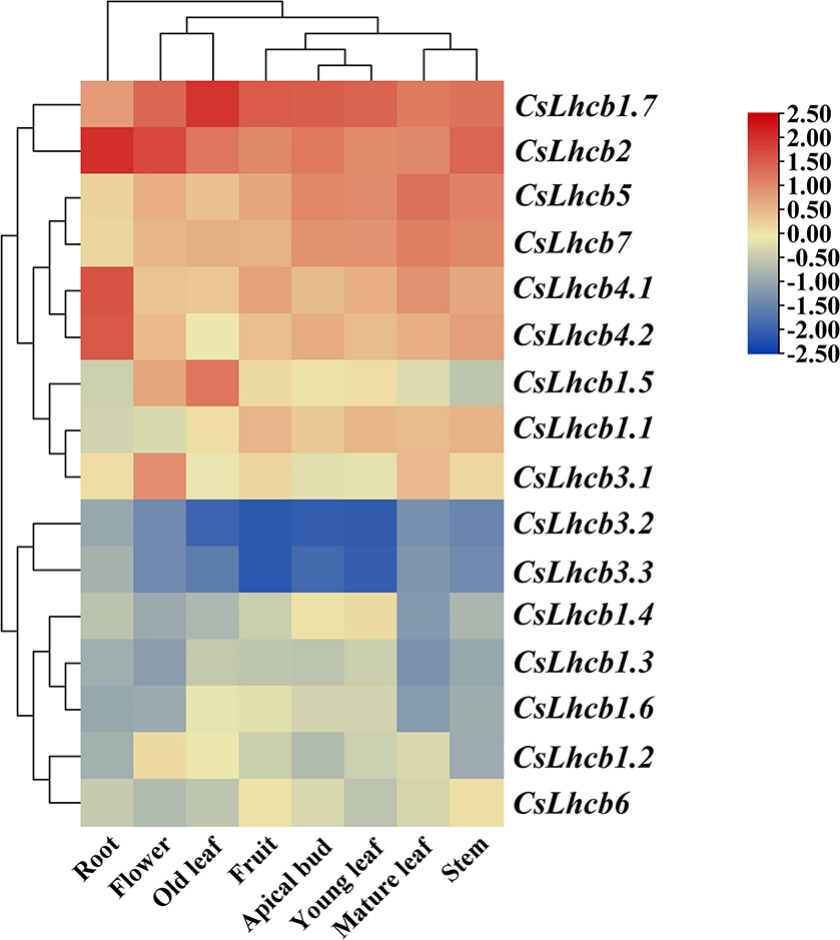
Expression profiles of *CsLhcb* family genes in different tissues of *C. sinensis*

In order to further understand the expression profiles of *CsLhcb* family genes in tea plant, TBtools software was used to make heat maps of the expression levels based on the transcriptome data (Fig. 5). The results showed that the expression levels of *CsLhcb1.7*, *CsLhcb2* and *CsLhcb5* were high in all tissues of tea plant. The expression levels of *CsLhcb3.2* and *CsLhcb3.3* were low in all tissues. In roots and flowers, the expression levels of *CsLhcb* gene family in tea plant was higher than that in other tissues, especially *CsLhcb2* gene. The expression levels of different genes in different tissues of tea plant were different, indicating that *CsLhcb* gene maybe involved in the growth process of different tissues of tea plant.

**Fig. 6:**
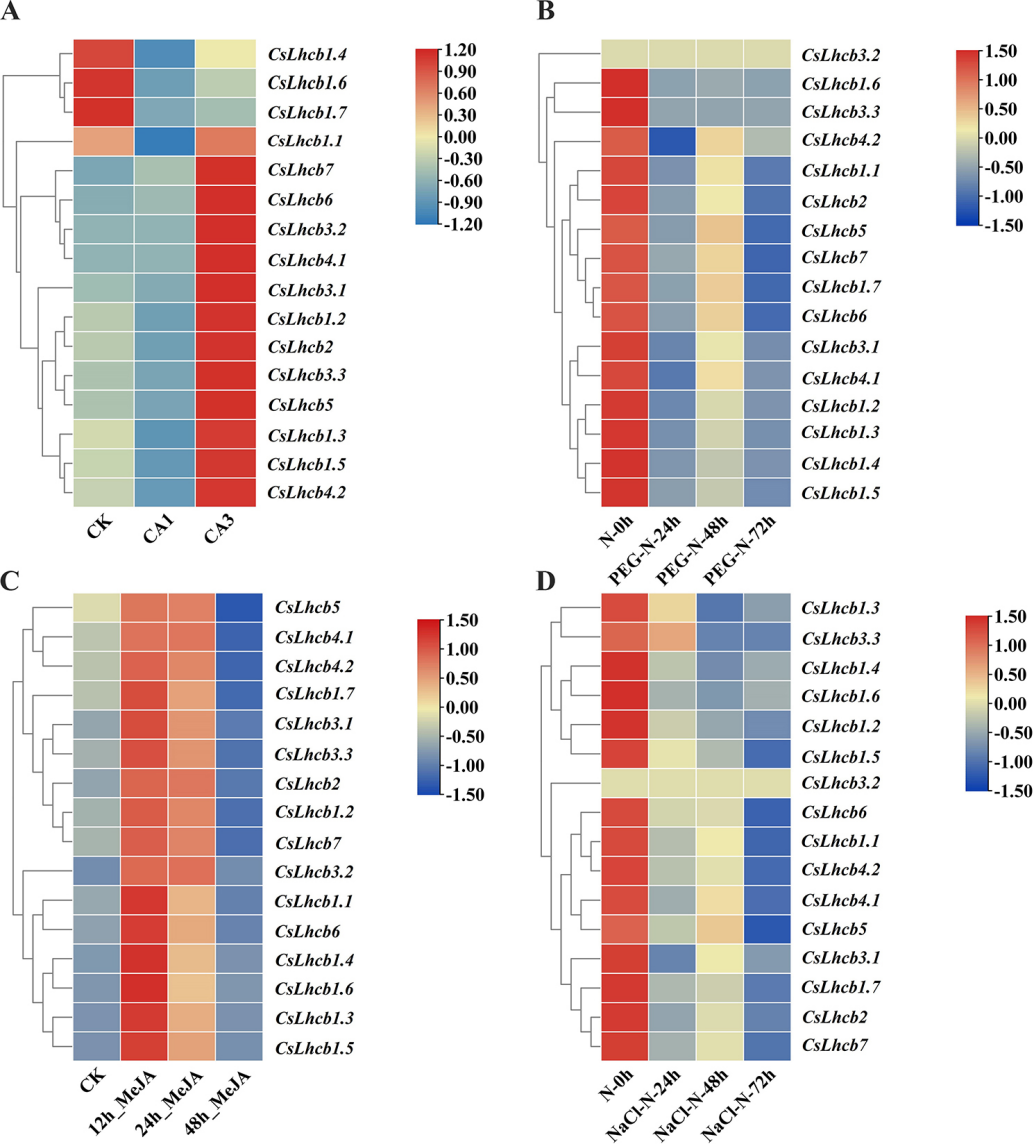
*CsLhcb* family genes expression profiles under different stresses in *C. sinensis ***A**. Cold treatment; **B**. Drought treatment; **C**. Methyl jasmonate treatment; **D**. Salt treatment

The *CsLhcb* genes profiles under abiotic stress treatments were also analyzed and heat maps were made (Fig. 6). Under cold stress, the expressions levels of *CsLhcb1.4*, *CsLhcb1.6* and *CsLhcb1.7* were decreased, while the expressions levels of the other 13 *CsLhcb* genes were increased. The expression levels of *CsLhcb* family genes decreased with the increase of time under drought stress and salt stress. The gene expression levels of *CsLhcb* genes were upregulated after 12 h methyl jasmonate treatment, and then, the levels decreased gradually with the increase of time.

### Analysis of photosynthetic parameters of circadian rhythm during two photoperiods in tea plants

**Fig. 7:**
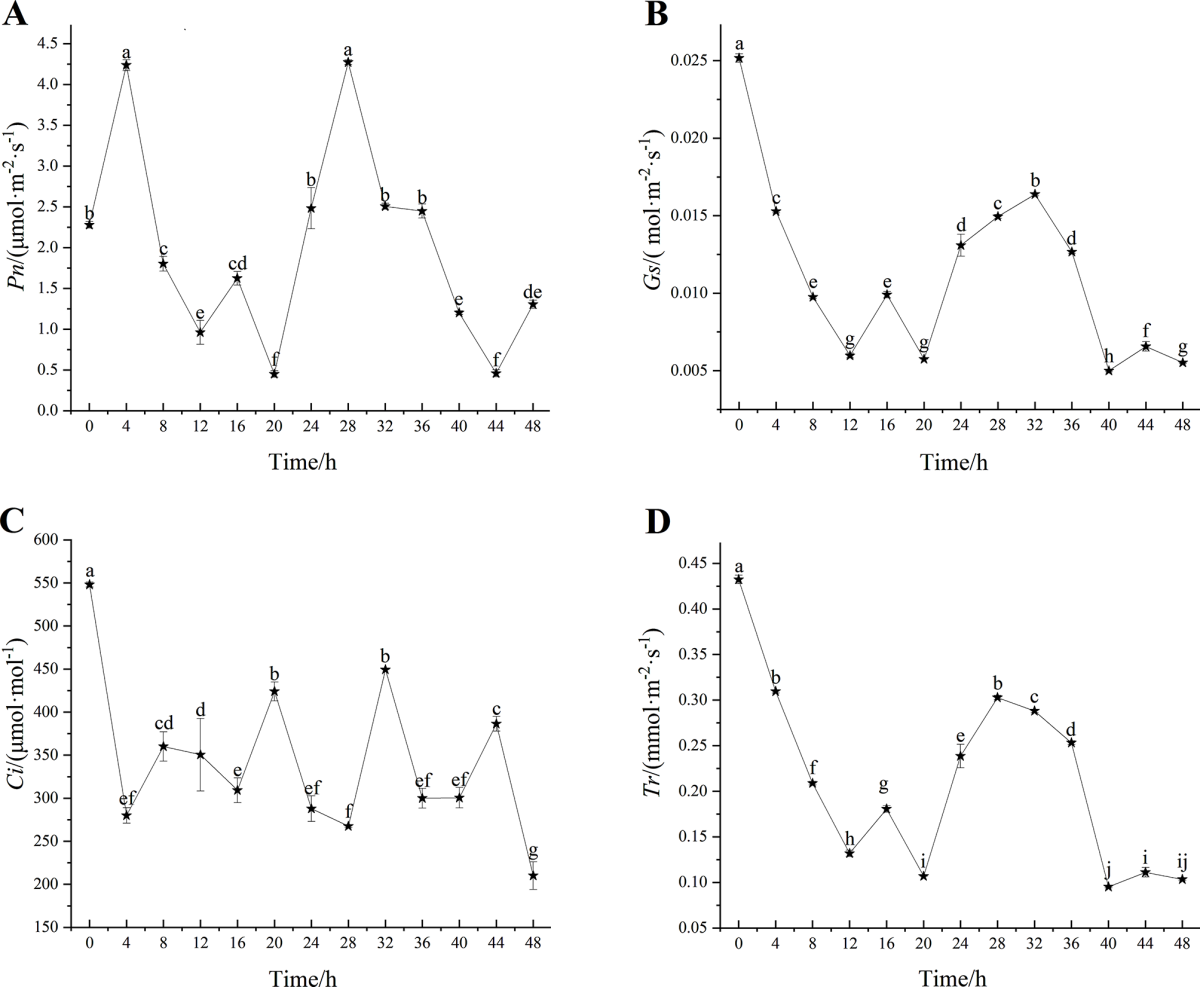
Analysis of photosynthetic parameters during the two photoperiod periods in *C. sinensis*

Net photosynthetic rate (*Pn*), stomatal conductance (*Gs*), intercellular CO_2_ concentration (*Ci*) and transpiration rate (*Tr*) were important parameters reflecting the photosynthesis of plant leaves. The circadian rhythm photosynthetic parameters of tea leaves were detected Interval of 4 h in two photoperiod periods (48 h) time periods (Fig. 7). The net photosynthetic rate (*Pn*) reached the peak at 4 h and 28 h, reaching the highest of 4.27 µmol·m^− 2^·s^− 1^, and then gradually decreased and reached the low point at night. The variation trends of stomatal conductance (*Gs*) and transpiration rate (*Tr*) were similar, and were significantly higher in daytime than at night. Intercellular CO_2_ concentration (*Ci*) decreased gradually from 0 h to 4 h, and then increased slowly, reaching the highest values at 20 h and 32 h, which were 423.96 mmol·mol^− 1^ and 449.35 mmol·mol^− 1^, respectively. The photosynthetic parameters of tea plant had little difference between the two photoperiods, which was in accordance with the rule of circadian clock.

### Analysis on the circadian rhythm of stomatal opening in tea leaves

**Fig. 8:**
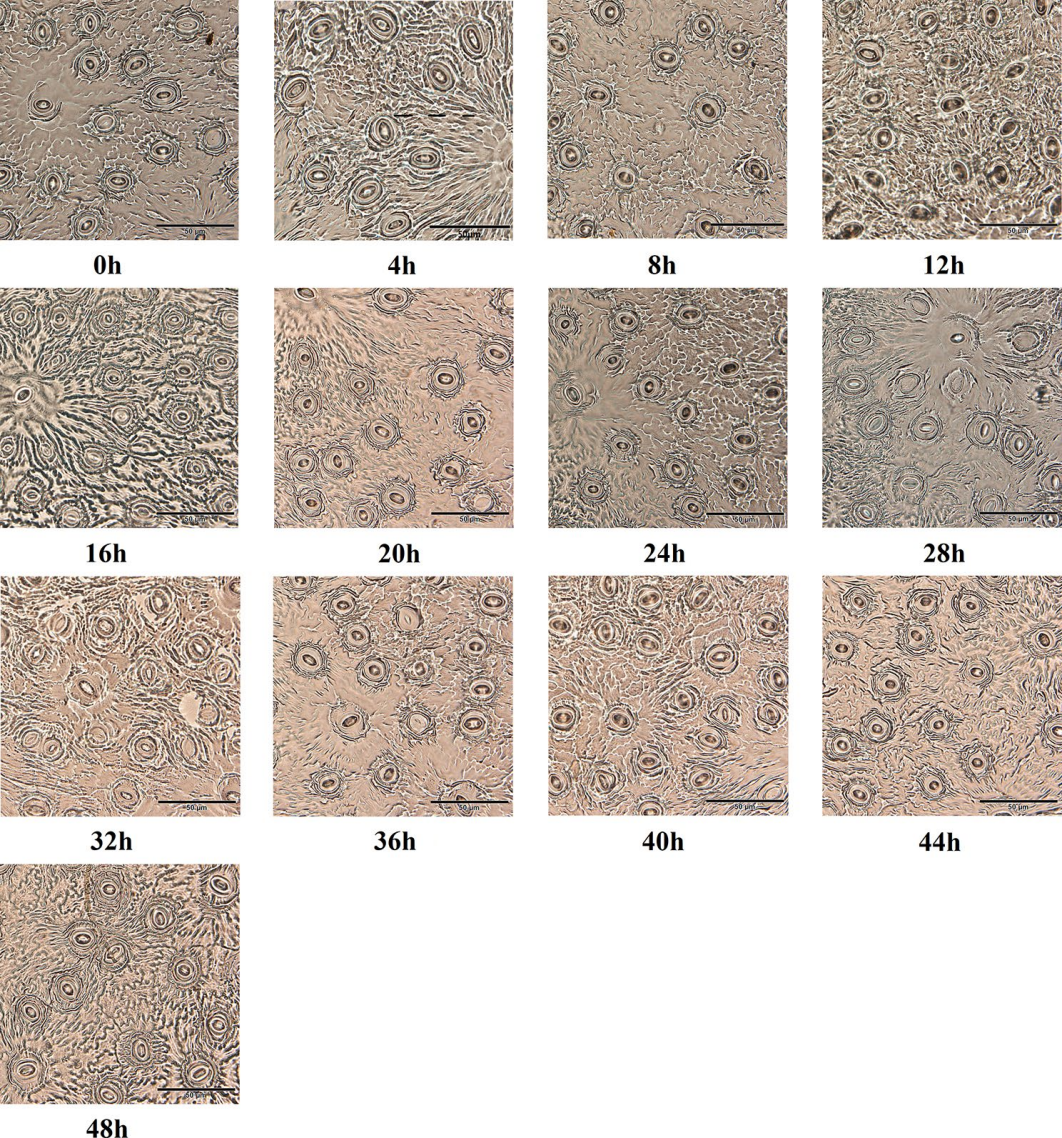
Analysis on the circadian rhythm of stomatal aperture in tea leaves

As shown in Fig. 8, stomatal openings of tea leaves were different at different time points. From 0 h to 4 h, the stomata opened slowly, and the opening gradually increased, 1.24 times of the initial value; At the 28 h, or the 4 h of the second photoperiod periods, the stomatal conductance of tea plant reached the peak of 61.95 µm^2^. Then the tea plant underwent a short ‘lunch break’ phenomenon, and the stomata slightly contracted compared with the previous stage. Stomatal conductance decreased gradually at night, and reached its lowest level of 33.69 µm^2^ at 20 h (44 h, second photoperiod periods) the next day (Table 2).

**Table 2:** *Cis*-acting element analysis of *CsLhcb* family genes in *C. sinensis*

Gene ID	*Cis* acting element										
	Light	Circadian	TGA-element	P-box	LTR	TCA-element	ABRE	ARE	CGTCA-motif	MBS	
*CsLhcb1.1*	***√***	***√***	**—**	***√***	**—**	***√***	**—**	***√***	**—**	**—**	
*CsLhcb1.2*	***√***	***√***	**—**	***√***	**—**	***√***	**—**	***√***	**—**	**—**	
*CsLhcb1.3*	***√***	**—**	**—**	***√***	***√***	**—**	**—**	**—**	**—**	**—**	
*CsLhcb1.4*	***√***	**—**	***√***	***√***	**—**	**—**	**—**	***√***	***√***	**—**	
*CsLhcb1.5*	***√***	**—**	**—**	***√***	**—**	***√***	**—**	***√***	**—**	***√***	
*CsLhcb1.6*	***√***	**—**	**—**	***√***	**—**	***√***	**—**	***√***	**—**	***√***	
*CsLhcb1.7*	***√***	**—**	**—**	**—**	**—**	**—**	***√***	***√***	***√***	***√***	
*CsLhcb2*	***√***	**—**	**—**	**—**	**—**	**—**	***√***	***√***	***√***	***√***	
*CsLhcb3.1*	***√***	**—**	**—**	**—**	***√***	***√***	**—**	***√***	***√***	***√***	
*CsLhcb3.2*	***√***	***√***	**—**	**—**	**—**	**—**	***√***	***√***	**—**	***√***	
*CsLhcb3.3*	***√***	***√***	**—**	**—**	**—**	***√***	***√***	***√***	***√***	***√***	
*CsLhcb4.1*	***√***	**—**	***√***	**—**	***√***	**—**	***√***	***√***	***√***	***√***	
*CsLhcb4.2*	***√***	**—**		**—**	**—**	**—**	***√***	***√***	***√***	**—**	
*CsLhcb5*	***√***	**—**	***√***	**—**	***√***	**—**	***√***	***√***	***√***	**—**	
*CsLhcb6*	***√***	***√***	***√***	**—**	***√***	**—**	***√***	***√***	***√***	***√***	
*CsLhcb7*	***√***	***√***	**—**	**—**	**—**	***√***	***√***	***√***	**—**	***√***	

### Changes in circadian rhythm of *CsLhcb* family genes expression in tea plant

**Fig. 9:**
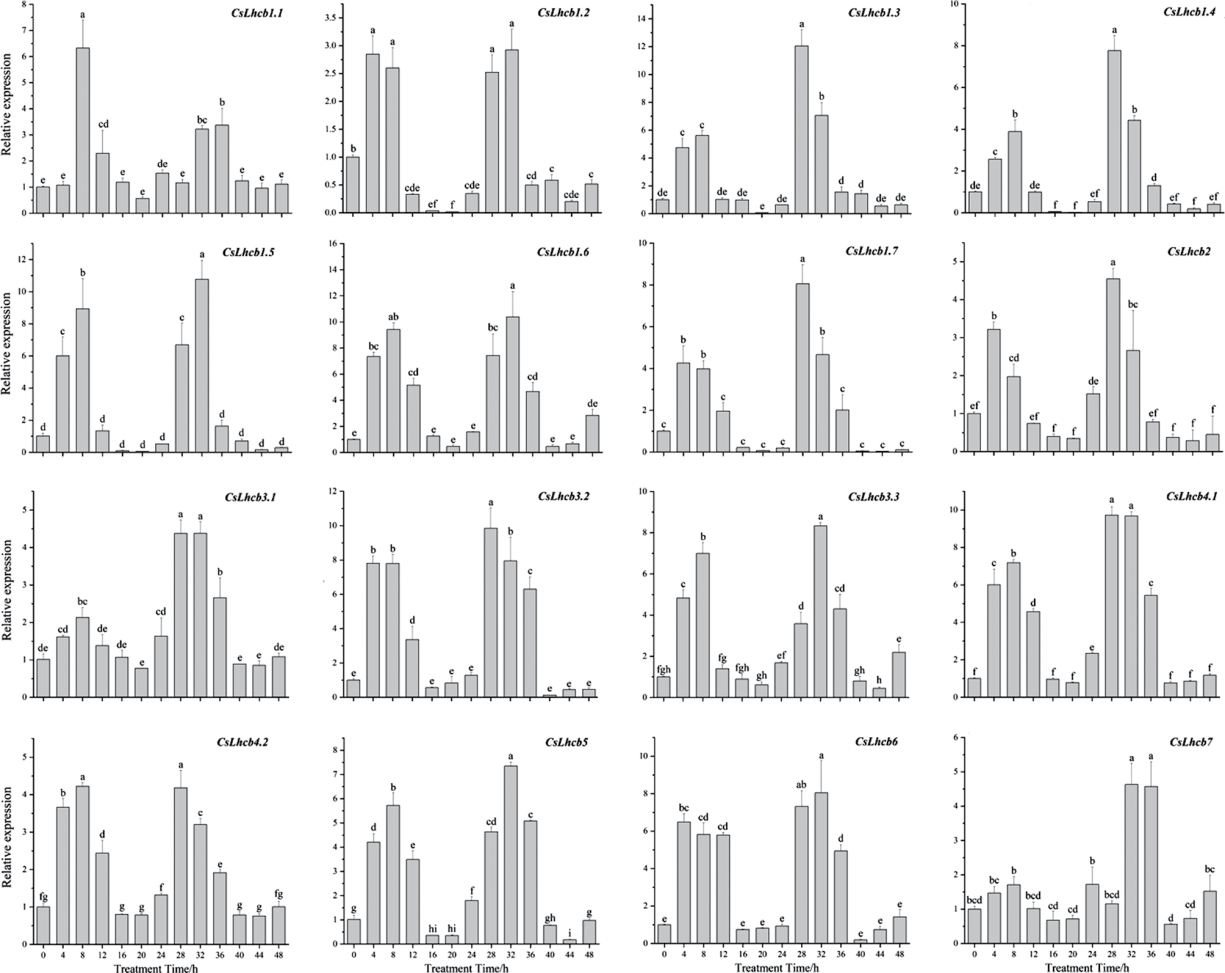
Circadian rhythm variation of *CsLhcb* family genes expression profiles in *C. sinensis*

The expression levels of *CsLhcb* genes increased first and then decreased during the two photoperiod periods, and were highly expressed during the daytime, especially at 1 pm and 5 pm during the two periods (0–24 h and 24–48 h), with the highest value reaching 12.05, which was *CsLhcb1.3* at 28 h. In the first photoperiod, some of the genes reached their maximum value after 8 h of continuous light, and only *CsLhcb1.2*, *CsLhcb1.7*, *CsLhcb2* and *CsLhcb6* reached their maximum value at 4 h. At night (1 am, 5 am), the expression levels were lower than that in day. The expression of some genes was trace expressed (Fig. 9). The trends of *CsLhcb* genes expression were consistent with circadian rhythm in tea plant.

## Discussion

In nature, the growth and development of plants are inseparable from light, light too strong or weak will have more or less influence on it. In the process of adaptation to the light environment, circadian clock genes play important roles in the adaptation of plants to the external light environment [4]. In previous studies, clock genes, such as *CCA1*, *LHY* and *TOC1*, have been reported in *Arabidopsis*, tobacco and *Phaseolus vulgaris* [27, 28], but there are few reports on circadian clock gene in tea plant [29, 30].

In this study, the *CsLhcb* family genes involved in circadian rhythm of tea plant was identified and analyzed in detail. The results showed that a total of 16 *CsLhcb* family genes were identified, while the number of family genes in rice and *A. thaliana* were 9 and 15, respectively. This suggests that members of the *CsLhcb* family have been Increased during the evolutionary process [31, 32]. Sixteen *CsLhcb* genes were distributed on 8 chromosomes of tea plant, which were located on chromosomes 1, 3, 4, 5, 7, 9, 10 and 12, respectively. Three homologous gene clusters were formed on the chromosomes of each *CsLhcb* member, indicating that there was a certain evolutionary relationship between the members. As an important functional gene in the optical system, *Lhcb* gene plays an important role in both light capture and transmission [33]. Through the prediction analysis of *cis*-acting elements in the promoter region of *CsLhcb* gene in tea plant, the results showed that light response elements were all present in *CsLhcb* gene, indicating that all members of *CsLhcb* family genes were involved in the light reaction process. There were also six genes involved in the photoperiod phase of the tea plant. This result was also consistent with effects of light and temperature on the expression of the *Lhcb2* gene in pea [34]. In addition to the widespread presence of *Lhcb* gene in light response, almost all genes also have anaerobically induced cis-acting elements. Deng et al. transferred the *LeLhcb2* gene into tobacco and overexpressed it, and found that the overexpression of *LeLhcb2* played a key role in reducing PSII photooxidation and enhancing the tolerance of transgenic tobacco to low temperature stress. These findings further confirm the role of this gene family in adaptation to environmental stress [35].

In higher plants, the content of chlorophyll in green plants is closely related to plant photosynthesis. The decrease of chlorophyll content will lead to the decrease of the photosynthetic rate, and even affect the yield of crops in severe cases [36]. The *CsLhcb* gene family plays a key role in chloroplasts [37]. Tea leaves growing under shady conditions contain higher chlorophyll content [38, 39]. Liu et al. found that two central signal integrators (GLK1 and LHCB) respond significantly to shade in ‘Shuchazao’, indicating that light played important roles in regulating chloroplast development in tea leaves, which is similar to *A. thaliana* [40]. By silencing the *AtLHCB1* gene, Pietrzykowska et al. resulted in slower growth of *A. thaliana*, smaller leaves, and reduced chlorophyll content [41].

Photosynthesis has a direct impact on the growth and development of tea plant. Here, the photosynthetic parameters of ‘Baiyeyihao’ were measured during the two photoperiod periods (48 h). The results showed that the values of *Pn*, *Gs* and *Tr* were higher in the daytime than at night, and the photosynthetic parameters of tea plant had little difference between the two photoperiods. which was in accordance with the rule of circadian rhythm. Meanwhile, the circadian rhythm of stomatal opening in tea leaves was also analyzed, and it was found that stomatal opening in daytime was significantly higher than that at night, and the variation of stomatal opening was also consistent with the expression level of *CsLhcb* gene family members. The changes of stomatal opening and *Gs* are similar, and the increase of stomatal opening plays a key role in improving the photosynthetic characteristics of plants [42]. In higher plants, stomatal pore size was one of many processes that regulate circadian rhythms. The opening and closing of stomata were driven by changes in the inflationary pressure of the guard cells. In *Arabidopsis*, stomata open early in the morning when ambient temperatures were low. Under field conditions, the circadian rhythm has a significant effect on the gas exchange at the leaf-canopy scale [43]. At the same time, several key genes in the circadian control pathway that regulates photoperiodic flowering have also been shown to influence stomatal aperture. Among them, GI (GIGANTEA) and CO (CONSTANS) up-regulated FT (FLOWERING LOCUS T) level leading to stomatal opening, while ELF3 (EARLY FLOWERING3) inhibited FT leading to stomatal closing [44, 45]. Hassidim et al. proved that photoperiodic pathway components CO and FT, which regulate flowering time, also regulate stomatal aperture in a way dependent on day length [46].

The expression level of *CsLhcb* genes increased first and then decreased during the two photoperiods, with high expression in daytime and low expression at night, suggesting that the *CsLhcb* genes were related to the circadian clock [47]. Millar et al. found that the expression of the *Lhcb* gene in *Arabidopsis* usually oscillates robustly during each daylight and dark cycle, with peaks and troughs observed during the light and dark phases, respectively. Rapid and brief ‘acute responses’ to light can be distinguished from peak clock regulation at dawn by good temporal resolution [22]. Setsuyuki Aoki et al. showed by Northern blotting that *Lhcb* mRNA levels in *Protosemitic* cells had a steady daily oscillation during the light-dark period (12:12 LD), and rapidly decreased during continuous darkness (DD) [48]. The *CmLhcb1* expression was higher in leaves than in stems, flowers, and roots. Low light and GA_3_ treatment increased the expression of *CmLhcb1*. In chrysanthemum, the expression of *CmLhcb1* is regulated by circadian rhythm, and the gene expression in daytime is significantly higher than that at night [49].

## Conclusions

In this study, members of the *CsLhcb* family genes of tea plant were identified and analyzed, the classification and evolutionary relationship were also determined. A total of 16 *CsLhcbs* genes were distributed on 8 chromosomes based on the genome database of tea plants. *CsLhcb* members formed three homologous genes clusters in the chromosome of tea plant, indicating that there was a certain evolutionary relationship between the members. At the same time, the response of *CsLhcb* genes to circadian rhythm of tea plant were detected and analyzed, and the results indicated that *CsLhcb* genes were involved in the circadian clock process of tea plant. It was found that the expression levels of *CsLhcb* genes in tea plant within 24 h was related to the trend of photosynthetic parameters, and was basically consistent with stomatal opening state. The results provided a potential research direction and basis for further study on the function of *CsLhcb* gene family and response to circadian rhythm in tea plant.

## Methods

### Plant materials and growth conditions

The cutting seedlings of two-year-old ‘Baiyeyihao’ were cultivated in State Key Laboratory of Crop Genetics & Germplasm Enhancement and Utilization (32° 04′ N, 118° 85′ E, Nanjing, China). The ‘Baiyeyihao’ were provided by the Tea Science Research Institute of Horticulture College, Nanjing Agricultural University. As an albino variant tea variety, ‘Baiyeyihao’ was often used in genetic breeding research. Healthy tea seedlings were selected and placed in the artificial climate chamber (RXZ-380, Jiangnan, Ningbo, China) for pre-culture for 2 days (temperature 25 °C, photocycle 12 h /12 h, light intensity 320 µmol·m^− 2^ s^− 1^, humidity 70–80%). The sampling was started at 9:00, and healthy tea seedlings were selected every 4 h within 48 h, and one bud and two leaves were picked, wrapped in tin foil and quickly frozen in liquid nitrogen for subsequent quantitative experiments. The second leaf at the top of the tea seedling was picked at each time point for the determination of stomata later. The freshly prepared 1 mmol· L^− 1^ methyl jasmonate solution was sprayed on the leaves of different treated plants for hormone treatment, and the leaves were collected after treatment (0, 12, 24, 48 h). The tea plants were watered with 20% sodium chloride solution and 20% PEG-6000 solution respectively (500 mL solution), and the leaves were collected after the two treatments (0, 24, 48, 72 h). Leaves were treated at 4 ℃ (0, 12, 24, 48 h) in a light incubator. At the same time, 5 plants with the same growth were randomly selected for the determination of photosynthetic parameters, and each plant was repeated for 3 times.

### Screening and identification of *Lhcb* gene family in tea plant

The TPIA database (http://tpia.teaplant.org/) was used to download tea protein group and the genome data. TAIR database (www.arabidopsis.org/index.jsp) was used to download *Arabidopsis* AtLHCB amino acid sequence. Then, the identified AtLHCB sequences were submitted to the Pfam database (http://pfam.sanger.ac.uk) and the resulting LHCB domain was represented by the Pfam entry number PF00504.24. The specific conserved domain of *Lhcb* gene was used for BLASTP alignment and the threshold was set as (*E*-value ≤ 10^− 5^) to obtain the amino acid sequence of CsLHCB in tea plant. CDD with NCBI database (https://www.ncbi.nlm.nih.gov/Structure/cdd/wrpsb.cgi) domain analysis verify CsLHCB protein structure, eliminate does not include the Chloroa-b-bind domain of protein structure.

### Construction of *CsLhcb* gene family phylogenetic tree

According to ClustalW, the LHCB protein sequences of *A. thaliana*, rice (*O. sativa*) and tea plant (*C. sinensis*) were submitted to MEGA7.0 software (version: 7.0.26) for sequence alignment, and then the evolutionary tree model was constructed using the software. The sequences of the OsLHCB family were downloaded from a database published by the Joint Genomics Institute. The specific method was neighbor joining analysis, the Boostrap parameter was set to 1000, and the remaining parameters were the default of the system to construct the complete phylogenetic tree [50]. Finally, iTOL online software (iTOL: Interactive Tree of Life (embl.de)) beautifies the evolutionary tree.

### Analysis of *CsLhcb* gene structure and protein conserved motifs of tea plant

The *CsLhcb* genomic DNA sequence and cDNA sequence of tea plant was submitted to the online software TBtools (version: 1.045) to draw the gene structure map. MEME (http://memesuite.org/) was used for conservative domain analysis, and the number of motifs was set to 10, and the width range was set to 6-50AA. The conserved motifs of LCHB protein were mapped using TBtools [51].

### Analysis of *cis*-acting elements of *CsLhcb* family genes in tea plant

The upstream 2000 bp sequence of *CsLhcb* gene start codon was obtained by TBtools software according to tea plant genome data. Then use the plant CARE software (https://bioinformatics.psb.ugent.be/webtools/plantcare/html/) to forecast the original *cis* elements *CsLhcb* gene family role, Finally, use Excel 2010 to make tables and analyze relevant data.

### Analysis of tissue pattern expression profiles of *CsLhcb* family genes in tea plant

The expression level of *CsLhcb* gene in different tissues of tea plant and the expression level of *CsLhcb* gene in tea plant under abiotic stress were downloaded from TPIA database (http://tpia.teaplant.org/). TBtools was used to map the expression of *Lhcb* family genes under different tissue and abiotic stress.

### RNA extraction and cDNA synthesis

The extraction of total RNA from tea plant samples was completed using the RNA Isolation Kit (Huayueyang, Beijing, China). The concentration of RNA samples was determined by micro ultraviolet detector Nanodrop ND-1000. The RNA quality was detected by 1.2% agarose gel electrophoresis. Total RNA extracted from tea leaves was reversely transcribed into cDNA using reverse transcription kit (TaKaRa, Dalian, China). The Primers for reverse transcription were Oligo (dT) Primer. The steps were 1 µL Oligo (dT) and 1 µL RNA were added, and the rest was filled with RNase-free water for a total of 12 µL in a water bath at 65℃ for 5 min. Then reverse transcription reagent was added into the reaction tube of the first step. The amplification program was set at Oligo (dT): 42 ℃ for 60 min, reverse transcription: 70 ℃ for 5 min, termination: 4 ℃. Store at -20 ℃ for later use. Three biological replicates were performed for each sample.

### Detection of leaf photosynthetic parameters

The leaf photosynthetic parameters were determined by LI-6400XT portable photosynthetic apparatus (LI-COR, Lincoln, Nebraska, USA). Net photosynthetic rate (*Pn*), stomatal conductance (*Gs*), intercellular CO_2_ concentration (*Ci*) and transpiration rate (*Tr*) of leaves were measured. The air velocity was 500 µmol·s^− 1^, light intensity was 600 µmol·m^− 2^ s^− 1^, temperature was (20 ± 1) ℃, relative humidity was 70 − 75%, and CO_2_ concentration was 400 µmol·mol^− 1^.

### Leaf stomatal section detection

While the light and parameters were measured, the second leaf on the top of the tea seedling was picked, the dust on the leaf surface was wiped off, and the transparent nail polish was evenly applied on the back. The leaf was dried for about 5 min, and then glued with transparent tape on the slide, samples were made and stored. A total of 3 biological replicates were performed. The preserved samples were observed with optical microscope Olympus BX-53 (Olympus, Japan) and measured their length and width. Finally, the stomatal opening of leaves was calculated. Stomatal opening (c)=πab, where a represents 1/2 pore length and b represents 1/2 pore width [52].

### RT-qPCR analysis

**Fig. 10:**
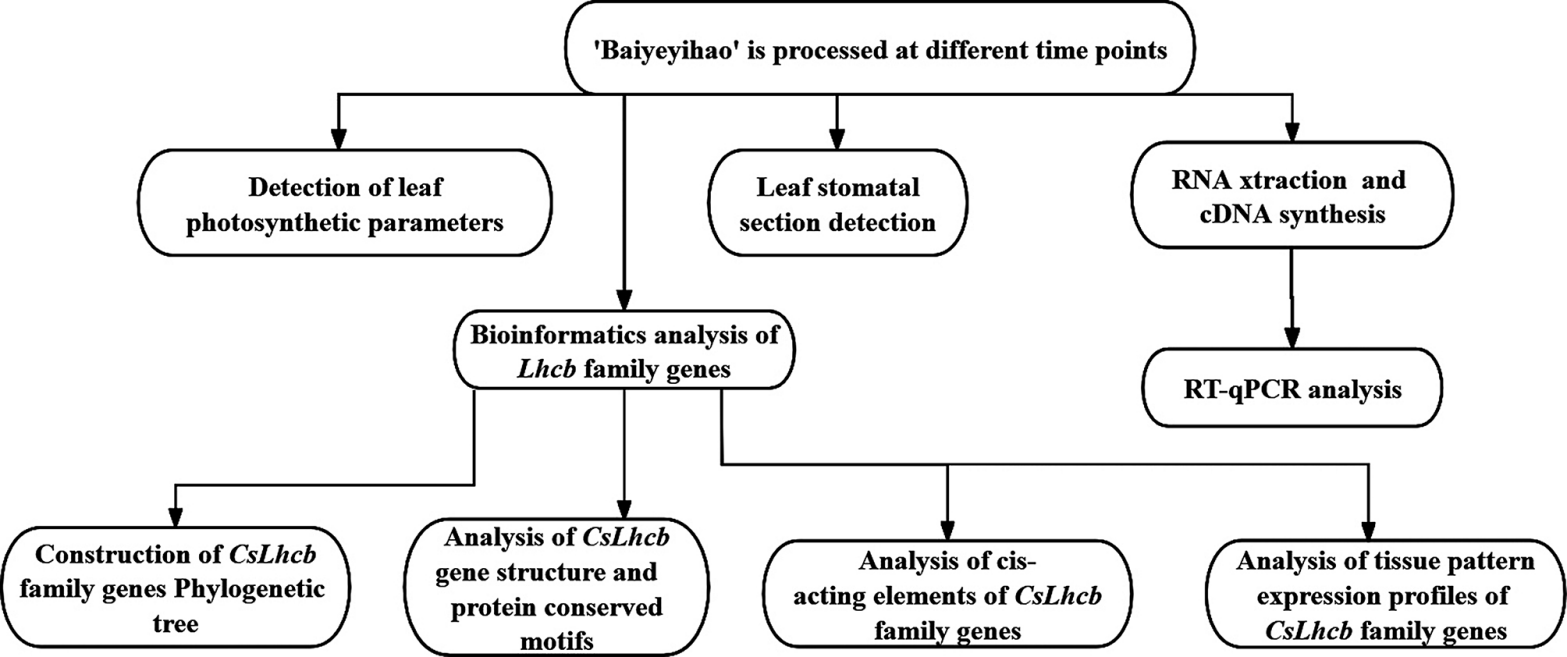
Flowchart of experimental method

We conducted a comprehensive analysis on the circadian rhythm changes of all 16 *Lhcb* family genes expression profiles, and detected the expression level every 4 h in tea plants during two photoperiods (48 h). RT-qPCR primers were designed using Primer Premier6.0 software. Real-time fluorescent quantitative PCR was based on the SYBR Premix *Ex Taq* Kit specification (TaKaRa, Dalian, China) and on the ABI7500 (Applied Biosystems, Foster city, USA). Tea plant *GAPDH* gene was selected as the internal reference gene, and the amplification primers were CsGAPDH-F and CsGAPDH-R [53]. The systems used for amplification were 20 µL: 10 µL SYBR Green I mix, 0.4 µL positive and negative fluorescent quantitative primers, 2.0 µL cDNA, 7.2 µL ddH_2_O. The amplification program was set at 95 ℃ for 5 min. Denatured at 95 ℃ for 10 s, annealed at 54 ℃ for 30 s, extended at 65 ℃ for 15 s, a total of 40 cycles. A total of three biological replicates were performed, and 2^−ΔΔCt^ was used to calculate the relative gene expression levels. The flowchart of the experimental method in this study was shown in Fig. 10. Primers for RT-qPCR of *CsLhcbs* genes and internal reference gene were listed in Table 3.

**Table 3:** Stomata aperture of tea leaves during the two photoperiod periods

Time/h	Stomatal longitudinal diameter/µm	Stomatal transverse diameter/µm	Stomatal aperture/µm^2^
0	9.43 ± 0.16	6.58 ± 0.16	48.69 ± 2.01
4	12.29 ± 0.29	6.25 ± 0.13	60.28 ± 2.56
8	10.75 ± 0.28	6.04 ± 0.13	51.00 ± 1.70
12	11.52 ± 0.11	6.19 ± 0.16	56.02 ± 0.96
16	10.60 ± 0.17	6.07 ± 0.11	50.52 ± 0.19
20	9.16 ± 0.31	5.08 ± 0.29	36.55 ± 3.34
24	10.00 ± 0.29	6.42 ± 0.31	50.42 ± 3.69
28	11.64 ± 0.16	6.78 ± 0.16	61.95 ± 2.23
32	10.74 ± 0.1	5.63 ± 0.14	47.52 ± 1.64
36	11.27 ± 0.23	5.89 ± 0.11	52.09 ± 1.85
40	10.23 ± 0.22	5.63 ± 0.14	45.25 ± 1.51
44	8.57 ± 0.28	5.01 ± 0.18	33.69 ± 1.19
48	10.49 ± 0.34	6.10 ± 0.26	50.27 ± 1.46

### Data processing and analysis

Microsoft Excel 2019 software was used for data sorting, the significance of differences was analyzed using IBM SPSS Statistics 25.0 (version: 25.0), Duncan’s was used for multiple comparisons (*P* < 0.05), and Origin 8.0 software was used to complete the graph production.

### Electronic supplementary material

Below is the link to the electronic supplementary material.

Supplementary Material 1

## Data Availability

The TPIA database (http://tpia.teaplant.org/) was used to download tea protein group and the genome data. TAIR database (www.arabidopsis.org/index.jsp) was used to download *Arabidopsis* AtLHCB amino acid sequence.

## Abbreviations

Ci: Intercellular CO_2_ concentration
Gs: Stomatal conductance
LD: Light-dark period
Lhcb: light-harvesting chlorophyll a/b-binding proteins
MeJAS: Methyl jasmonate
Mw: Molecular weight
pI: Isoelectric point
Pn: Net photosynthetic rate
RT-qPCR: Real-time quantitative PCR
Tr: Transpiration rate
